# Biosynthesis and Cellular Functions of Tartaric Acid in Grapevines

**DOI:** 10.3389/fpls.2021.643024

**Published:** 2021-03-04

**Authors:** Crista Ann Burbidge, Christopher Michael Ford, Vanessa Jane Melino, Darren Chern Jan Wong, Yong Jia, Colin Leslie Dow Jenkins, Kathleen Lydia Soole, Simone Diego Castellarin, Philippe Darriet, Markus Rienth, Claudio Bonghi, Robert Peter Walker, Franco Famiani, Crystal Sweetman

**Affiliations:** ^1^Agriculture and Food, CSIRO, Glen Osmond, SA, Australia; ^2^School of Agriculture, Food and Wine, The University of Adelaide, Urrbrae, SA, Australia; ^3^King Abdullah University of Science and Technology, Thuwal, Saudi Arabia; ^4^Division of Ecology and Evolution, Research School of Biology, The Australian National University, Acton, ACT, Australia; ^5^Western Barley Genetic Alliance, Murdoch University, Perth, WA, Australia; ^6^College of Science and Engineering, Flinders University, Bedford Park, SA, Australia; ^7^Wine Research Centre, Faculty of Land and Food Systems, The University of British Columbia, Vancouver, BC, Canada; ^8^Université Bordeaux, Unité de recherche OEnologie, EA 4577, USC 1366 INRAE, Institut des Sciences de la Vigne et du Vin, Villenave d’Ornon, France; ^9^University of Sciences and Art Western Switzerland, Changins College for Viticulture and Oenology, Nyon, Switzerland; ^10^Department of Agronomy, Food, Natural Resources, Animals and Environment, University of Padova, Legnaro, Italy; ^11^Dipartimento di Scienze Agrarie, Alimentari e Ambientali, Università degli Studi di Perugia, Perugia, Italy

**Keywords:** grape, fruit, tartaric acid, metabolism, gene, enzyme, antioxidant

## Abstract

Tartaric acid (TA) is an obscure end point to the catabolism of ascorbic acid (Asc). Here, it is proposed as a “specialized primary metabolite”, originating from carbohydrate metabolism but with restricted distribution within the plant kingdom and lack of known function in primary metabolic pathways. Grapes fall into the list of high TA-accumulators, with biosynthesis occurring in both leaf and berry. Very little is known of the TA biosynthetic pathway enzymes in any plant species, although recently some progress has been made in this space. New technologies in grapevine research such as the development of global co-expression network analysis tools and genome-wide association studies, should enable more rapid progress. There is also a lack of information regarding roles for this organic acid in plant metabolism. Therefore this review aims to briefly summarize current knowledge about the key intermediates and enzymes of TA biosynthesis in grapes and the regulation of its precursor, ascorbate, followed by speculative discussion around the potential roles of TA based on current knowledge of Asc metabolism, TA biosynthetic enzymes and other aspects of fruit metabolism.

## Introduction

L-Tartaric acid, (2,3-Dihydroxybutanedioic acid, L-threaric acid, TA) accumulates in comparatively few plant species despite its close structural similarity to many other C_4_-dicarboxylates. A product of the oxidation of sugars, in many cases via L-ascorbate (Asc) breakdown, it appears to have no known physiological or biochemical function. In cultivated crops the accumulation of TA is largely non-responsive to stresses or environmental or cultural management practices. A five or six-step synthesis pathway was proposed almost 40 years ago, and whilst candidate enzymes have been characterized for two steps, little is understood about how, where or indeed why this relatively simple compound accumulates in the tissues of just a few plant species (DeBolt et al., 2007).

Scientific interest in TA would likely have been minimal were it not for the happy circumstance that it is the principal acid found in berries of the cultivated grapevine *Vitis vinifera* (Kliewer, 1966). At harvest, there are 4 to 8 grams of TA per L of grape juice, contributing in large part to a pH of 2.9 to 3.8 (Amerine et al., 1965). TA is thereby responsible for much of the ‘vitality’ of wine, balancing out the inherent sweetness of alcohol on the palate and contributing to low pH conditions needed to enable the wine to age over an appropriate period whilst minimizing spoilage from microbial and oxidative processes (Plane et al., 1980; Zoecklein et al., 1995; Liu et al., 2007).

Notwithstanding its economic and cultural value, important questions remain unanswered about TA. The most widely accepted TA synthesis pathway begins with Asc, although precisely how Asc is converted to 2-keto L-gulonate, the first intermediate in the pathway, remains unknown. Some suggest that the oxidized form of Asc, dehydroascorbate (DHA; a product of ROS scavenging), serves as the precursor (Hancock and Viola, 2005; Melino et al., 2009a). Either way, it would seem an unusual fate for Asc, more commonly referred to as vitamin C, which is better known for its role in regulating the cellular redox state, among other metabolic and signaling processes (Gilbert et al., 2009; Smirnoff, 2011; Foyer et al., 2020). None of the intermediate compounds in the pathway are commonly found in plants, nor do they have roles in alternative pathways. It is therefore to be wondered why five or six enzymes have been retained in grapevine for the sole function of producing a compound with no known role, from a compound with several enormously important roles.

It is of course likely that we are yet to elucidate its function, a fate which for many plant compounds has previously seen them allocated to the category of ‘secondary metabolite’. More recent definitions have revised this classification to ‘plant specialized metabolites’, with the defining characteristics of such compounds being that they are (i) not directly involved in growth or development, (ii) restricted to a narrow set of species, (iii) not necessary for survival and (iv) support stress responses. Additionally, specialized metabolites are classified as either phenolics, alkaloids or terpenes. TA does not fit this latter requirement and may therefore be proposed as a ‘specialized primary metabolite’, being a product of essentially primary (i.e., carbohydrate) metabolism, but with none of the energy conservation, biosynthetic or regulatory functions normally associated with these biomolecules.

Trending decreases in titratable acidity and increases in pH of grape juice have been observed over time and in response to increased temperatures or other climatic effects (Spayd et al., 2002; de Orduña, 2010; Sadras et al., 2013; Godden et al., 2015). These can lead to the need for acid additions to restore appropriate pH and TA levels if wine quality is to be preserved. This is often a significant expense to winemakers, particularly during warmer growing seasons. In the broader food industry, TA can be used as an additive for its acidic and antioxidant properties (Silva and Lidon, 2016; EFSA Panel on Food Additives and Flavourings et al., 2020). Unlike TA, malic acid (MA), which also accumulates in grapes during early development, is susceptible to enzymatic catabolism during ripening, particularly at high temperatures (Buttrose et al., 1971; Kliewer, 1971; Ruffner et al., 1976). Potassium levels in grape berries can increase with temperature due to increased water uptake, thus exacerbating the effect on pH that arises from the exchange of protons from the vacuole during cation transport (Boulton, 1980; Coombe, 1987). With increased pH of juice and must (the mixture of juice, skins and seeds that is fermented to make red wines), larger SO_2_ additions may be required to effectively curb the growth of undesirable microbes. This can lead to wines exceeding legal SO_2_ thresholds, also affecting wine flavor and aroma (de Orduña, 2010). Several decades of work has explored acidity in winegrapes, including the variation of acidity measured among different *Vitis* species (Stafford, 1959; Kliewer et al., 1967; Shiraishi et al., 2010; reviewed by Dai et al., 2011), *V. vinifera* cultivars (Shiraishi, 1995; Liu et al., 2007) and within populations (Liang et al., 2011; Viana et al., 2013; Duchêne et al., 2014; Chen et al., 2015; Houel et al., 2015; Ban, 2016), as well as the metabolic effects of temperature on organic acids in grapes (Kliewer, 1973; Ruffner et al., 1976; Sweetman et al., 2014).

The aim of this review is to summarize new developments in the area of TA accumulation and biosynthesis in grape berries, although some parallels are drawn with other fruits. Furthermore, potential roles of TA in fruit metabolism are proposed, drawing on what is now known of the biosynthetic enzymes and precursors.

## Tartaric Acid Accumulation in Grape Berries (Concentration and Distribution)

In fruit, TA was once thought to be uniquely accumulated in grapes, but it has since been shown to occur at significant levels in a range of other fruits, including avocado (Pedreschi et al., 2019), lychee (Wang et al., 2006), sweet cherry (Mahmood et al., 2012), blueberry (Li et al., 2015), tamarind (Van den Bilcke et al., 2014), some citrus fruits (Nour et al., 2010) and in one report, banana (Wyman and Palmer, 1964). A tabulated summary is available in a recent review on fruit organic acids (Walker and Famiani, 2018). TA has also been measured in leaves of bean (Saito and Loewus, 1989), tamarind (Lewis and Neelakanthan, 1959), geranium and grapevine (Williams and Loewus, 1978). Recent technological advances have enabled very accurate and high-throughput quantification of organic acids in individual grapes (Melino et al., 2009b; Higginson et al., 2016), revealing large variation in TA concentrations between bunches within a vine and between berries of a single bunch, likely due to asynchronous development of the individual fruit.

Net TA accumulation is exclusive to the first stage of berry development, associated with rapid cell division (Kliewer and Nassar, 1966). Asc shares an almost identical developmental accumulation pattern although at approximately one-fiftieth the concentration (Melino et al., 2009a; Cholet et al., 2016). TA biosynthesis reactions are thought to occur in the cytosol of plant cells (Pignocchi et al., 2003), however the proposed involvement of transketolase and succinate semialdehyde dehydrogenase in late steps (Salusjärvi et al., 2004; DeBolt et al., 2006), as well as a protein localization study of the L-idonate dehydrogenase (Wen et al., 2010), discussed in later sections, suggest that additional cellular compartments and even the apoplast may be involved in grape TA biosynthesis. Once synthesized, TA resides in the vacuole (DeBolt et al., 2004).

Accumulation of TA occurs in all *V. vinifera* cultivars and across the Vitaceae family (Stafford, 1959). TA levels in *V. vinifera* fruit are largely unaffected by environmental conditions other than in a small handful of studies that report responses to light (Melino et al., 2011; Reshef et al., 2017), water deficit (Grimplet et al., 2009; Savoi et al., 2017), fertilization with silicon and calcium chloride (Gomes et al., 2020), grafting onto different rootstocks (Zhang et al., 2020b) and seasonal variability due to water status and light exposure (Cholet et al., 2016). Notwithstanding these reports, there is no evidence that any one or combination of cultural practices can be used to modulate levels of TA in berries at harvest.

The search for QTLs relating to grape berry acidity has seen a lot of activity in the last decade, with some promising breakthroughs for total or titratable acidity (Liang et al., 2011; Duchêne et al., 2014; Ban, 2016), pH (Viana et al., 2013; Chen et al., 2015), MA (Chen et al., 2015; Duchêne et al., 2020) and various acid ratios in young berries (Houel et al., 2015), however in most cases there was no satisfactory marker for TA level (Liang et al., 2011; Viana et al., 2013; Chen et al., 2015; Bayo-Canha et al., 2019). This may be due to the involvement of several biochemical steps and potential sites of regulation, as well as other factors that affect berry acid levels such as potassium accumulation (Duchêne et al., 2020). Despite these challenges, two major QTLs for TA concentration on linkage groups LG7 and LG4 were stably detected using a Picovine x Ugni Blanc flb population under different environmental conditions (Houel et al., 2015). Most of the above QTL analyses were based on mapping within bi-parental populations, which may be restricted by the limited genetic diversity between the specific parental varieties or species. Therefore, the selection of appropriate crossing parents may be critical for the success of TA QTL studies. In addition, genome-wide association studies (GWAS) that exploit the great genetic and phenotypic diversities of large grapevine germplasm collections deserve more attention for the study of complex biological traits such as TA accumulation. This approach is becoming increasingly feasible for woody species like grapevine (c.f. cereal crops), due to the lower costs of high-throughput sequencing and phenotyping techniques. Recently, GWAS analyses of 472 *Vitis* accessions (Liang et al., 2019) and 279 *V. vinifera* L. cultivars (Flutre et al., 2020), were employed to identify loci associated with traits including TA accumulation, detailed further in Section 9.

## Biosynthetic Enzymes of Tartaric Acid

**FIGURE 1:**
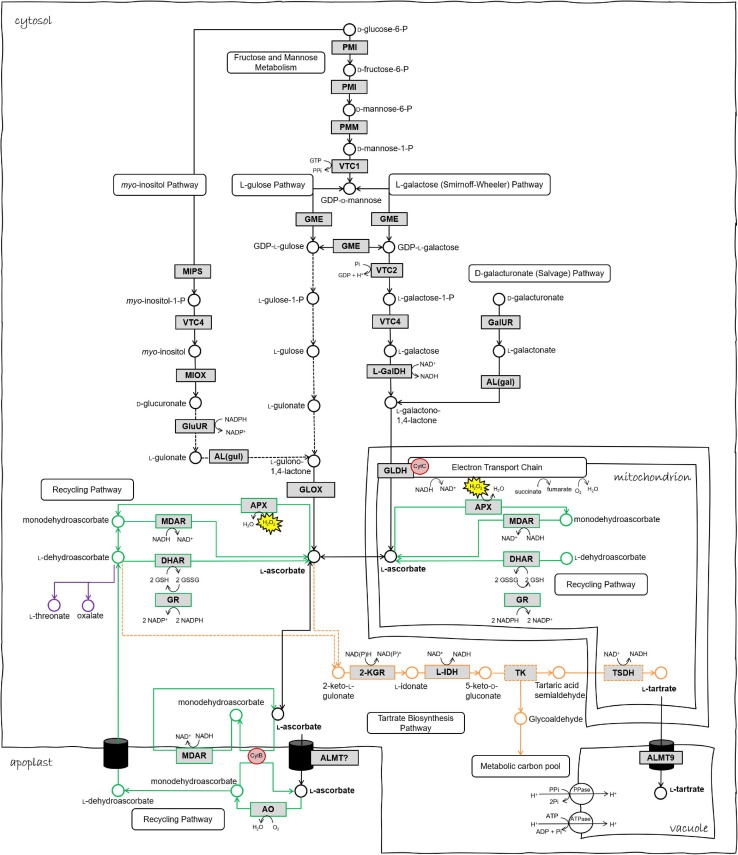
Biosynthetic and redox pathways of ascorbic acid and tartaric acid in the plant cell. Chloroplastic ascorbate redox reactions are omitted from this schematic. Black arrows/metabolites/enzymes represent ascorbate biosynthesis steps. Green represents ascorbate recycling pathways in various cell compartments. Orange represents tartrate biosynthesis pathway. Dashed arrows or boxes indicate hypothetical reactions or enzymes. Enzyme abbreviations as follows. PMI phosphomannose isomerase, PMM phosphomannomutase, VTC1 GDP-D-mannose pyrophosphorylase, GME GDP-D-mannose epimerase, VTC2 GDP-galactose phosphorylase, VTC4 L-galactose-1-phosphate phosphatase, L-GalDH L-galactose dehydrogenase, GLDH L-galactonolactone dehydrogenase, MIPS *myo*-inositol-3-phosphate synthase, MIOX *myo*-inositol oxidase, GluUR D-glucuronic acid reductase, AL(gul) aldono-lactonase for L-gulonate, GLOX L-gulonolactone oxidase, GalUR D-galacturonic acid reductase, AL(gal) aldono-lactonase for L-galactonate, MDAR monodehydroascorbate reductase, DHAR dehydroascorbate peroxidase, APX ascorbate peroxidase, GR glutathione reductase, AO ascorbate oxidase, 2KGR 2-ketogulonic acid reductase, L-IDH L-idonate dehydrogenase, TSDH tartaric acid semialdehyde dehydrogenase, TK transketolase, ALMT aluminum-activated malate transporter, cytB Cytochrome B561. Figure adapted from Melino et al. (2009a), Burbidge (2011), and Podgórska et al. (2017).

Three pathways of TA biosynthesis occur in higher plants, each identified by the precursor and cleavage site, namely “Asc C4/C5,” “Asc C2/C3,” and “D-gluconic acid C4/C5,” (Loewus, 1999), the former being the primary pathway in grape and described in detail by Ford (2012). Briefly, in the Asc C4/C5 pathway (depicted in Figure 1), Asc is converted by one or more uncharacterized steps to 2-keto L-gulonate and thence via reduction to L-idonate and oxidation to form 5-keto D-gluconate. This six-carbon intermediate is cleaved by an unknown enzyme to yield a four-carbon intermediate, possible tartaric acid semialdehyde that is finally oxidized to produce TA. In the Asc C2/C3 pathway, Asc is cleaved between carbons two and three: the two-carbon fragment forms oxalic acid and the four-carbon fragment forms L-threonic acid which is subsequently oxidized to form TA. The D-gluconic acid C4/C5 pathway, which has been identified in leguminous species, has a direct conversion of D-gluconic acid to 5-keto-D-gluconic acid via an unidentified oxidation step, whereafter the formation of TA is thought to occur by the same steps as proposed for the Asc C4/C5 pathway. The sequence of redox-associated steps in the confirmed reactions of TA synthesis, which may feature also in the conversion of Asc to 2-keto L-gulonate and in the steps leading from 5-keto D-gluconate, has been suggested to reflect a common strategy in the generation of phytochemical diversity (Horn, 2021).

By feeding labeled Asc to immature berries of a number of species from the Vitaceae family it was determined that [1-^14^C]Asc and [4-^14^C]Asc corresponded to the C1 and C4 carbons respectively of the TA skeleton (Williams and Loewus, 1978). Earlier studies that identified the key intermediates and organization of the C4/C5 pathway (Saito and Kasai, 1969; Wagner and Loewus, 1974; Wagner et al., 1975) were complemented by later work identifying the final intermediates (Saito and Kasai, 1982, 1984). Accumulation of radiolabeled L-idonate in grapevine leaves also fed with labeled Asc confirmed that the oxidation of L-idonate to 5-keto-gluconic acid is a rate-limiting step of TA synthesis (Malipiero et al., 1987).

Non-targeted metabolomics studies in grape berries provide opportunities to look for accumulation of other intermediates under a range of conditions, however our own search of published metabolomics datasets for the purpose of this review, turned up little evidence. One report showed developmental and temperature regulation of ketogluconate levels (Gouot et al., 2019), but it is unclear which isomer(s) were present in the analysis. Although the steps of the “Asc C4/C5” pathway were identified decades ago, further work on the biosynthesis of TA in grape berries and identification of the enzymes responsible has been limited (Ford, 2012), but some recent findings warrant summarizing.

### L-Idonate Dehydrogenase

L-idonate dehydrogenase (L-IDH) catalyzes the above-mentioned “rate-limiting” step of L-idonate to 5-keto-gulonic acid (Figure 1) and was the first enzyme of the TA biosynthetic pathway to be identified and characterized (DeBolt et al., 2006). In *V. vinifera* there are three L-IDH isoforms, which are co-located on chromosome 16 but only one (*VvLIDH3*; Q1PSI9; VIT_16s0100g00290) has been shown definitively to oxidize L-idonate (DeBolt et al., 2006; Sweetman et al., 2012; Higginson et al., 2016). *VvLIDH1* (ABA01327) has a very high sequence identity to *VvLIDH3* with only three like-for-like (e.g., hydrophobic-for-hydrophobic or neutral) amino acid substitutions at non-critical sites (Higginson, 2015; Jia, 2015). *VvLidh1* and *VvLidh3* transcripts both show preferential expression in young fruit (Deluc et al., 2007; Melino et al., 2009a; Sweetman et al., 2012; Rienth et al., 2014; Massonnet et al., 2017), thus aligning with the timing of TA accumulation. This may be seasonally dependent in some cultivars such as Trincadeira (Fortes et al., 2011), although it is unclear whether the microarray used in this study could discriminate between the three *VvLidh* isoforms. Data from another study using microarray data and potted vines in climate chambers suggest that *VvLidh3* may be up-regulated at night in ripening berries (Rienth et al., 2014).

Development of an antibody to L-IDH (*VvLIDH3* peptide was used as a target but the antibody was not checked for cross-reactivity against the other isoforms) and an enzyme assay compatible with berry extracts enabled the first observations that the L-IDH protein and its activity peak during early berry development, similar to transcript levels of both *VvLidh1* and *VvLidh3*, but linger during ripening (Wen et al., 2010, 2014; Cholet et al., 2016). Given the persistence of the protein beyond the period of TA accumulation, post-translational mechanisms such as protein degradation or sequestration of the enzyme by vacuolar autophagy (Wen et al., 2010), feedback inhibition (Cholet et al., 2016), or the availability of precursors (Melino et al., 2009a, 2011) may explain the cessation of TA accumulation at veraison. Wen et al. (2010) suggested that L-IDH continues to function in the berry vacuole during ripening: if so, it must occur at a low rate, with optimal pH of the recombinant enzyme between pH 8 and 9 and a five-fold drop in specific activity at pH 7 (DeBolt, 2006; Jia, 2015).

Berries of the wild Chinese grapevine *Ampelopsis aconitifolia* lack *Lidh* transcripts and TA (DeBolt et al., 2006), suggesting that L-IDH is essential for TA biosynthesis. Wen et al. (2014) showed that the transcript levels of *Lidh* (in this case a combination of *VvLidh3* and *VvLidh1* orthologs, as it is difficult to design unique primer sets) did not correlate with berry TA levels across *Vitis* species but instead, a high TA accumulator had a slower decline of the L-IDH protein throughout ripening as compared to a lower TA-accumulator. However, it is important to factor in berry size, which can differ significantly across species and cultivars, exerting a dilution effect on the TA content of berries. This warrants further analysis of the data by Wen et al. (2014). In one of the first successful CRISPR/Cas9 mutagenesis studies in grapevine, disruption of *VvLidh3* led to a decrease, but not a total loss of TA in Chardonnay cell cultures (Ren et al., 2016). The authors suggested that residual TA synthesis could be symptomatic of the type of mutation (i.e., no frameshift) or due to a combination of wild type and transgenic cells present within the cell mass. Complementary activity from the *VvLIDH1* isoform might also explain this, unless the guide RNA could not discriminate between both copies and thereby both isoforms were edited within the same cell.

L-IDH enzymes have recently been classified as “Class II” plant sorbitol dehydrogenases (SDH), with a set of key amino acid residues (His42, Gly112, and Ser113) likely responsible for the evolutionary diversification from sorbitol to L-idonate substrate binding and oxidation (Jia et al., 2015). These key residues are conserved in *VvLIDH1* and *VvLIDH3* but not *VvLIDH2*, which is instead designated as a “Class I” SDH and likely involved in sorbitol metabolism rather than TA biosynthesis. Residues predicted to be important for L-idonate selectivity have also been highlighted in the L-IDH ortholog of the TA-accumulating geranium (Narnoliya et al., 2018). The expression patterns of the Class I and Class II SDHs of grapevine also differ, with the former increasing throughout development and the latter aligning more closely with the timing of TA biosynthesis, i.e., decreasing throughout development (Sweetman et al., 2012; Jia et al., 2015; Cholet et al., 2016). *VvLidh1* and *VvLidh3* are genetically linked at 10 kb apart, in the same orientation on chromosome 16, whereas *VvLidh2* is also situated nearby but in the opposite orientation (Higginson, 2015; Jia et al., 2015), suggesting regulation by different promoters.

In apple and sweet orange, the presence of putative L-IDH orthologs did not equate to TA accumulation, leading to some doubt as to the importance of L-IDH in TA biosynthesis (Shangguan et al., 2015). Upon closer inspection of the apple genome, 11 of the 12 potential orthologs had low sequence similarity to the confirmed *VvLIDH3* and importantly, they lacked the key residues identified by Jia et al. (2015) and Narnoliya et al. (2018). That is, only one copy of a Class II plant SDH is present in apple and sweet orange (Jia et al., 2015) and the Class I SDHs may not catalyze the L-idonate oxidation reaction. The only “Class I” SDH to be tested for L-IDH activity, COC280 (Uniprot: A0A061FZU3) from *Theobroma cacao* did not display any convincing oxidation of L-idonate despite its close homology to *VvLIDH3* (Jia, 2015). Therefore, the enzymatic function of these L-IDH orthologs remains undetermined. With respect to the lack of TA in apple and sweet orange, low expression of proteins responsible for upstream steps of the pathway could also conceivably deprive these fruits of L-idonate precursors, an avenue yet to be explored. Therefore L-IDH remains the best-characterized enzyme of the grape berry TA biosynthesis pathway.

### 2-Keto-L-Gulonic Acid Reductase

Efforts to identify enzymes responsible for other steps of the TA biosynthesis pathway have proven challenging, however some progress has been made toward the 2-keto-L-gulonic reductase (2-KGR; VIT_09s0002g04300), which precedes the L-IDH step (Figure 1). A grape gene with homology to the *Escherichia coli* 2-KGR from Yum et al. (1998) showed the same developmental expression pattern as *VvLidh3* (Burbidge, 2011). The recombinant enzyme had 2-KGR activity, however substrate affinity kinetics favored the enzyme as a glyoxylate or hydroxypyruvate reductase that may have additional activity as a 2-KGR (Burbidge, 2011; Jia et al., 2019). Indeed, in plants the grape *Vv2KGR* has the highest sequence similarity with the cytosolic *Arabidopsis thaliana* hydroxypyruvate reductase isoform 2 (*AtHPR2*), which acts as a compensatory bypass for hydroxypyruvate and glyoxylate reduction (Timm et al., 2008). However, recombinantly expressed *Vv2KGR* was also active, albeit far less efficiently, with L-idonate (i.e., the reverse reaction) as well as Asc, formate, sorbitol, D-glucose, 6-phosphogluconate, 5-keto-D-gluconate and D-gluconate (Burbidge, 2011). Subsequently, the protein structure of *Vv2KGR* was resolved using x-ray crystallography, and molecular docking studies revealed 2-keto-L-gulonate as the optimal substrate, while GC-MS confirmed the product of this reaction as L-idonate (Jia et al., 2019). Based on *in vitro* studies this enzyme may be considered a 2-KGR that has been commandeered from another metabolic pathway. Both *Vv2KGR* and *VvLIDH3* retain significant “original” enzyme activities, i.e., HP/glyoxylate reduction and sorbitol oxidation, respectively (Jia, 2015; Jia et al., 2019). This is a common observation for gene evolution via duplication, in which the newly duplicated genes evolve novel functions while still retaining their original functions (Flagel and Wendel, 2009).

### Intracellular Transporters

Once synthesized, TA is translocated to the vacuole for storage. An aluminum-activated malate transporter (ALMT) protein, encoded by *VvAlmt9* (XM_002275959), is likely to shuttle both TA and MA into the vacuole (Patel, 2008; Rongala, 2008; De Angeli et al., 2013). It is expressed throughout berry development but highest in ripe fruit according to transcriptomic data from Shiraz berries (Sweetman et al., 2012) and qPCR data from Aragonez berries (De Angeli et al., 2013), therefore its activity is likely to remain important long after net accumulation of MA and TA.

At present there are no candidates for TA transport at any other membrane of the plant cell but there are some candidates for transport of the precursor, Asc. There are twelve candidate genes for nucleobase ascorbate transporters (NAT) in Arabidopsis, three of which (*AtNat7*, *8* and *12*) are localized to the plasma membrane and at least one in the thylakoid membrane. However, it is unknown if these transport Asc or nucleobases (Maurino et al., 2006; reviewed by Foyer et al., 2020). A chloroplast envelope transporter of Asc (*AtPht4:4*) has also been identified (Miyaji et al., 2015), and chloroplast uptake is dependent on membrane potential and cytosolic Asc concentration. There is little understanding of the mechanisms by which Asc travels from its site of synthesis in the mitochondrion throughout the berry cell.

## TA Catabolism

Endogenous TA in the grape berry is not catabolized at any appreciable rate (Ruffner, 1982). Instead, it is widely accepted that the acid forms a stable salt of potassium bitartrate, which is sequestered in the vacuole away from potential catabolizing enzymes and thereby essentially untouched throughout ripening (Takimoto et al., 1976; Moskowitz and Hrazdina, 1981; Ruffner, 1982; DeBolt et al., 2004). However, degradation of exogenously applied radiolabeled TA could be recovered as CO_2_ from excised grape berries, suggesting that TA-catabolizing enzymes do exist in *V. vinifera* (Hrazdina et al., 1984). *E. coli* and *Pseudomonas* spp. can use D-tartrate as a carbon source via oxidation to oxaloacetate or glycerate and eventually pyruvate (Vaughn et al., 1946; Dagley and Trudgill, 1963; Kohn and Jakoby, 1968). L-tartrate, the isomer present in grapes, can be used for carbon fixation by *Agrobacterium vitis*; a bacterium that harbors genes for TA-metabolizing enzymes and is hosted by *V. vinifera* (Otten et al., 1995). L-tartrate from grape must can also be catabolized by *Botrytis cinerea*, resulting in several different organic acids including malate, pyruvate, acetate, oxalate and oxaloacetate (Shimazu et al., 1984). Therefore, while the berry is intact, TA remains sheltered from catabolism; however, upon its release from the vacuole via mechanical damage (i.e., during harvest and crush), or upon pathogen invasion, TA likely becomes susceptible to catabolic enzymes from such microorganisms.

## Ascorbate as a Precursor for TA Biosynthesis

In plants, Asc is amongst the major vital antioxidants and fulfils a plethora of functions in different cellular components (Smirnoff, 2011; Foyer et al., 2020). For example, Asc acts as an enzyme cofactor and modulator of enzyme activity in the thylakoid membrane (Müller-Moulé et al., 2004), a reducing agent in the chloroplast (Krieger-Liszkay et al., 2008), a substrate for ethylene biosynthesis (Mirica and Klinman, 2008), and has roles in regulation of cell expansion, fruit-ripening and softening in the apoplast (Green and Fry, 2005; Gilbert et al., 2009). Physiological roles of Asc in plants also include defense (Conklin et al., 1996), growth and development (Dowdle et al., 2007), hormone and pathogen responses (Pastori et al., 2003) and programmed cell death (de Pinto et al., 2006). The redox couple Asc to DHA can influence the cellular redox state, which may be an important component in ROS signaling (Foyer and Noctor, 2005; Noctor, 2006).

Asc does not accumulate to high quantities in grapes compared to some other fruits, for example, ripe Shiraz grapes contain approximately 0.7 μmol.g^–1^ FW (Melino et al., 2009b) which is significantly lower than ripe strawberries (3.37 μmol.g^–1^ FW) and kiwifruits (3.41 μmol.g^–1^ FW) (Davey et al., 2000). It is not known for certain whether low Asc accumulators have a lower rate of Asc biosynthesis or an increased turn-over capacity, although *A. aconitifolia* berries, which do not accumulate TA, contain 3-fold more ascorbate compared to *V. vinifera* cv. Cabernet Sauvignon (DeBolt et al., 2006). Therefore, TA may primarily function to catabolize excess Asc, as recently proposed by Cholet et al. (2016) and blocking the TA biosynthetic pathway may increase vitamin C content in *V. vinifera* berries. Considering that there is no sudden accumulation of Asc at the conclusion of net TA biosynthesis in grapes, but there is reduced transcription of Asc biosynthetic genes in the Smirnoff-Wheeler pathway, a halt in the biosynthesis of Asc is a likely contributor to the plateau in TA levels from veraison (Melino et al., 2009a).

## Dehydroascorbate as a Precursor for TA Biosynthesis

Ascorbate peroxidase (APX) catalyzes the two-electron oxidation of Asc, reducing hydrogen peroxide (a potent ROS that can inactivate CO_2_-fixation enzymes) to water and forming DHA (via monodehydroascorbate, MDHA) in numerous cellular compartments. Asada (1992) described the value of regenerating reduced Asc as two-fold: to maintain capacity to donate electrons for the reduction of hydrogen peroxide and to protect against inactivation of APX.

In very young grape berries (10 days after flowering), over 70% of ^14^C from exogenously applied Asc was recovered within TA (Saito and Kasai, 1969). Generally, the reduced form of Asc is considered the fundamental precursor of TA in the cytosol due to its vast predominance over oxidized forms in the cell (Smirnoff, 2018). However, the cellular location of TA biosynthesis has not yet been definitively shown. Radiolabelling studies may largely represent apoplastic events due to the methods of radiolabel application (Smirnoff, 2018) and in the apoplast the Asc pool is rapidly oxidized to DHA (Pignocchi et al., 2003). Hancock and Viola (2005) proposed DHA as an important intermediate of TA biosynthesis; also labeled a “branch-point” for Asc catabolism by Parsons et al. (2011). When fed to grapevine leaf apices, radiolabeled DHA led to 2KGA, L-idonate and ultimately TA in a similar manner to radiolabeled Asc (Saito and Kasai, 1984). This suggests DHA as an intermediate in the conversion of L-Asc to 2KGA, a reaction that at the present time remains to be characterized. DHA catabolism to oxalic acid (OA) and other products in the culture media of *Rosa* cells suggest that such Asc catabolism can occur via DHA in the apoplast (Green and Fry, 2005). In grapes, OA and TA both accumulate within the same cells and demonstrate strikingly similar developmental accumulation profiles that show highest biosynthesis at a time when ascorbate is predominantly in the oxidized form (DeBolt et al., 2004; Melino et al., 2009a). During ripening, when net TA accumulation no longer occurs, there is a gradual shift to the reduced form (i.e., increased Asc to DHA ratio), likely due to decreased transcription of key genes responsible for Asc biosynthesis and increased transcription of genes responsible for recycling Asc from its oxidized forms (Melino et al., 2009a). There is also a substantial increase in the concentration and reduction state of glutathione (Adams and Liyanage, 1993; Okuda and Yokotsuka, 1999), which may redirect DHA toward Asc recycling and away from catabolic processes. Further correlative support arises through different grapevine tissues: roots have a small and highly reduced ascorbate pool (Asc to DHA ratio of 13.4) and accumulate little or no TA as compared to berries, rachis and leaves which have an Asc to DHA ratio of 1.1, 1.9, and 2.6 respectively (Kliewer, 1966; Melino et al., 2009b).

## Potential Roles of TA in Grape Berries Based on Predecessors of TA Biosynthetic Enzymes

### An Incidental Route to TA Accumulation?

As new enzymes of the TA biosynthesis pathway are uncovered, it will be interesting to see if these too are adapted from, or closely related to orthologs from other metabolic pathways, as proposed for L-IDH and 2-KGR. If so, TA accumulation could be a consequence of unrelated biochemical phenomena: high expression of enzyme isoforms that primarily catalyze other reactions but have adapted to recognize TA precursors as substrates, and where low affinity can be overcome by high precursor concentration (Melino et al., 2009a; Jia et al., 2015, 2019). That is, enzymes from other metabolic pathways could become “hijacked” when DHA or Asc levels are high, provided a particular (as yet unidentified) set of primary metabolic pathways are operating. This “incidental” route of TA biosynthesis might explain the narrow distribution of TA in the plant kingdom and the specific developmental pattern of accumulation in grape berries. Even the ALMT responsible for TA uptake into the vacuole (Terrier et al., 1998; De Angeli et al., 2013) could have been adapted from an exclusively MA transport function. Such evolution of a metabolic pathway is not unheard of in plants and may not mean that the pathway is unimportant: consider the glycolate pathway, adopted by modification of probably existing enzymes, now occurring in at least three organelles and cytosol, and essential in dealing with oxygenic photosynthesis (reviewed by Fernie and Bauwe, 2020).

From what we have garnered of grape berry TA biosynthetic enzymes so far, one enzyme (*Vv2KGR*) probably diverged from, or shares functionality with, a hydroxypyruvate or glyoxylate reductase (Jia et al., 2019) while another (*VvLIDH3*) diverged from a sorbitol dehydrogenase (Jia et al., 2015). The functions of these precursor enzymes will be explored below, to speculate on potential roles or reasons for TA biosynthesis in grape berries. Importantly, TA is also synthesized in grapevine leaves, likely by the same C4/C5 cleavage pathway found in grape berries (Williams and Loewus, 1978). Therefore, the roles of these divergent or promiscuous enzymes (outside of TA biosynthesis) are likely to be common between young berries and leaves. A role in photosynthesis or photorespiration seems most likely.

### A Sink for Ascorbate – The Hydroxypyruvate/Glyoxylate Reductase Alternative

Hydroxypyruvate reductase catalyzes the reversible conversion between hydroxypyruvate and glycerate in the peroxisome accompanied by the oxidation of NADH to NAD^+^. This reaction is essential during carbon recovery in photorespiration (Fernie and Bauwe, 2020). Glyoxylate reductase converts glyoxylate to glycolate in the cytosol, oxidizing NADPH to NADP^+^, and probably participates in the removal of toxic glyoxylate leaked from peroxisomes (Zhang et al., 2020a). Interestingly, these two activities arise from the same enzyme, along with 2-KGR activity. Considering the proposed dual roles of 2-KGR in reactions related to photorespiration, early reports of increased TA levels in light-exposed grape berries (Saito and Kasai, 1969; DeBolt et al., 2006) would seem to support a function for this enzyme in its synthesis. However, further investigation of berries grown for extended periods in light-excluding boxes suggested that the regulation by light applies also to the biosynthesis of Asc and that the transcription of *VvLidh3* was unaffected by light (Melino et al., 2011). These observations complicate the story, the light-mediated effects on ascorbate probably act to provide antioxidant buffering when ROS-generating photosynthetic activities are high. Only immature grape berries are capable of photosynthesis (Ollat and Gaudillère, 2000), coinciding with the timing of TA accumulation, which may therefore act as a sink for excess Asc as recently proposed elsewhere (Cholet et al., 2016). However, while the over-exposure of ripening berries to light caused depletion of Asc and glutathione levels, likely due to oxidation (DHA was not measured), there was no effect on TA levels (Rustioni et al., 2020).

### An Osmolyte – The Sorbitol Dehydrogenase Alternative

SDHs oxidize sorbitol and other sugar alcohols such as xylitol and ribitol to their corresponding monosaccharides (i.e., fructose, xylulose, and ribulose), reducing NAD(P)^+^ to NAD(P)H in the cytosol (Aguayo et al., 2013). Sorbitol is the main form of translocated sugar in some plants (mostly Rosaceae), though *V. vinifera* transports predominantly sucrose instead, and does not accumulate sorbitol to a high level (Swanson and El-Shishiny, 1958). In water-deficit conditions, grape berry SDH activity is down regulated, allowing sorbitol levels to increase (Conde et al., 2015). Knockdown of SDH in Arabidopsis led to improved tolerance to drought stress, likely due to osmo-protection from the higher levels of sorbitol (Aguayo et al., 2013). TA has also been suggested to enhance osmotic potential, especially in pre-veraison berries (Bigard et al., 2018) where it can be responsible for over 50% of total berry osmolarity, followed by MA at approximately 25% (Diakou et al., 1997). In the fruits of *Citrus reticulata*, *C. sinensis* and *C. paradisi*, treatment with proline (a stress-induced amino acid and osmo-protectant) led to decreased levels of TA, H_2_O_2_, and MDA (a lipid peroxidation product indicative of oxidative stress), as well as decreased lipoxygenase activity and increased APX activity (Mohammadrezakhani et al., 2019). In this case, excess proline may have reduced the need for other osmoprotective or ROS-protective mechanisms such as TA biosynthesis. Support for an osmo-protectant role of TA in grape and other fruits is merely correlative but warrants a targeted investigation.

## Potential Roles of TA in Grape Berries Based on Precursors of the TA Biosynthetic Pathway

### An Incidental or Dedicated Route to TA Accumulation?

**FIGURE 2:**
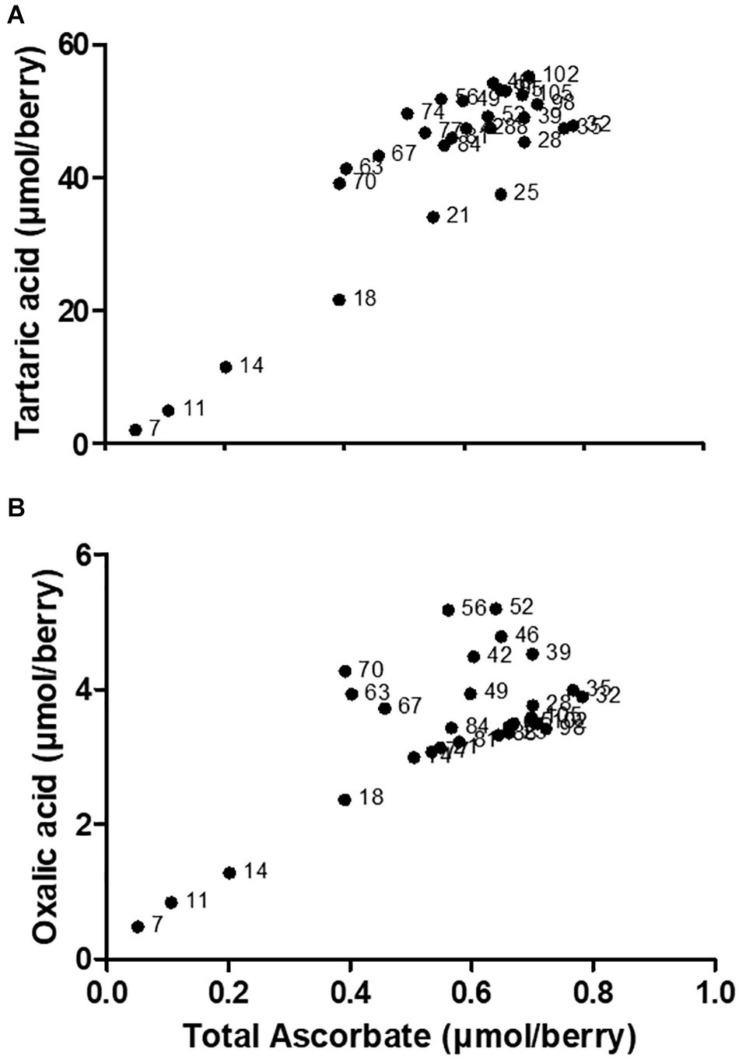
Correlation between **(A)** tartaric acid and **(B)** oxalic acid, with total ascorbate across *V. vinifera* c.v. Shiraz berry developmental series. Data points are labeled with physiological stage of berry (days after flowering). Correlation analysis was conducted using GraphPad Prism 5.01. The spearman correlation coefficients (rs) are 0.6486 and 0.4466, respectively. Adapted from Melino (2009).

In young grape berries a significant proportion of Asc is present as DHA (i.e., a low Asc to DHA ratio), which is engaged in the biosynthesis of OA and TA (Melino et al., 2009a). There is a strong positive correlation between the accumulation of total ascorbate (the sum of reduced and oxidized forms), and TA or OA in young berries (Figure 2). A regulatory link between total ascorbate levels and *VvLidh3* transcription has been proposed, and it follows that TA biosynthesis could be a storage mechanism for excess Asc or DHA under certain conditions (Cholet et al., 2016), generating an osmolyte that is sequestered in the vacuole during times of high Asc biosynthesis. Such a role is also consistent with the substantial accumulation of TA in grapevine leaves (Ruffner, 1982). This may support the hypothesized “incidental” route of TA biosynthesis, whereby the availability of precursors (i.e., Asc or DHA) is the limiting factor.

### Apoplastic Redox State, ROS Signaling and Cell Wall Softening

Additional roles for TA in grape berries may be found in the apoplast where it co-exists with other organic acids including Asc, OA and MA, as well as sugars and cations such as potassium (Wada et al., 2008; Keller and Shrestha, 2014; Podgórska et al., 2017). In leaves, the apoplast can contain up to 10% of the total cellular Asc content but this is highly oxidized due to the activity of ascorbate oxidase (AO), localized to the cell wall (Pignocchi et al., 2003; Karpinska et al., 2018). Although AO activity is enhanced by photosynthetic O_2_ (De Tullio et al., 2007), excessive AO activity can impair photosynthesis (Karpinska et al., 2018). In tobacco, high AO activity also led to the accumulation of apoplastic threonate, a DHA degradation product (Karpinska et al., 2018). The genome of *V. vinifera* (cv Pinot Noir) contains an unusually large number of genes encoding AO (9 copies) and APX (12 copies) compared to Arabidopsis (3 and 6 copies, respectively) and sweet orange (2 and 5 copies, respectively). Transcript levels of at least three AO genes (*VvAO1*, VIT_06s0009g01320; *VvAO3*, VIT_07s0031g01010; and *VvAO4*, VIT_07s0031g01040) followed a consistent developmental down-regulation in the grape berry (Fasoli et al., 2012; Sweetman et al., 2012; Savoi et al., 2017), analogous to the pattern of TA accumulation. In young *V. vinifera* berries with their multitude of AO genes and highly oxidized ascorbate pool, the accumulation of TA (instead of, or in addition to threonate) in the presence of light and photosynthetic O_2_, could provide an avenue for DHA degradation, in an attempt to balance the redox state of the apoplastic Asc pool.

The redox state of the apoplastic Asc pool is involved in ROS signaling (reviewed by Podgórska et al., 2017; Karpinska et al., 2018; Smirnoff, 2018). Signal transmission from the environment requires that some of the apoplastic Asc is kept in an ‘active’ (reduced) state (Pignocchi and Foyer, 2003), while an oxidized apoplastic Asc pool (i.e., high DHA) can inhibit photosynthesis and cell division but promote cell elongation and cell wall loosening (Pignocchi and Foyer, 2003; Pignocchi et al., 2003; Sanmartin et al., 2003; Karpinska et al., 2018). In tomato fruit, at the beginning of ripening Asc is excreted from the cell to the apoplast where it can generate hydroxyl radicals by reacting with H_2_O_2_ in the presence of ascorbate peroxidase, facilitating non-enzymatic degradation of polysaccharides in the cell wall and thus cell expansion (Dumville and Fry, 2003; Green and Fry, 2005). On the other hand, upon entry into the cell as a signal from the apoplast, DHA can slow cell cycle and proliferation in tobacco cell cultures (de Pinto et al., 1999; Potters et al., 2000). Reduction of DHA to Asc can apparently only occur in the cytosol, likely due to the lack of NADH and NADPH in the apoplast (Pignocchi and Foyer, 2003). Therefore, a specific transporter that can exchange apoplastic DHA for cytosolic Asc (Horemans et al., 2008), could simultaneously facilitate inhibition of cell division and enhancement of cell expansion – hallmarks of fruit ripening. It follows that TA biosynthesis could postpone cell wall loosening by catabolizing Asc (or DHA) in the apoplast. Based on transcriptomic datasets, higher transcription of *VvLidh3* may be associated with the firmer berries of Red Globe compared to soft berries of Muscat Hamburg (Ma et al., 2020). This transcript was also upregulated in berries afflicted with shrivel compared to non-shriveled berries co-existing on the same Zweigelt vines (Savoi et al., 2019). From veraison, phloem unloading into the berry switches from a symplastic route to apoplastic (Zhang et al., 2006), flooding the apoplast with high concentrations of hexoses and other solutes, potentially interrupting TA biosynthesis at the cell wall. Changes in apoplastic solutes affect cell turgor and berry firmness associated with veraison and necessary for ripening (Wada et al., 2008; Keller and Shrestha, 2014; Rogiers et al., 2017), as also seen in other fruits (Canton et al., 2020).

Although TA synthesis could take place in either or both cytosol and apoplast, further work is needed to confirm the apoplast as a site of synthesis. If a significant proportion of TA is synthesized in the apoplast there must be a transport mechanism for entry into the cell, to enable accumulation in the vacuole. Identification and characterization of this, and other transporters of the grape plasmalemma will help to determine DHA import kinetics and a mechanism for TA import, if it exists.

### Antioxidant Metabolism and the Oxidative Burst

ROS, antioxidants and their interactions are key to regulating fruit developmental stages and ripening (Muñoz and Munné-Bosch, 2018; Decros et al., 2019). Despite being classified as a non-climacteric fruit, grapes exhibit an oxidative burst in the skin cells as the berries begin to soften but prior to color change (Pilati et al., 2007). The ROS in this oxidative burst accumulate as superoxide in the chloroplast and hydrogen peroxide in the cytosol (Pilati et al., 2014). Hydrogen peroxide, when applied to bunches of young berries can also advance ripening (Guo et al., 2019). The mechanism by which hydrogen peroxide accumulates in the cytosol is unknown but a loss of catalase activity is unlikely to explain it (Pilati et al., 2014). Most likely it originates from another organelle and is transported to the cytosol via aquaporins (Bienert et al., 2006; Smirnoff and Arnaud, 2019).

Hydrogen peroxide accumulation may be a side-effect from the removal of superoxide from the chloroplast or from the loss of photosynthetic functionality. Alternatively, hydrogen peroxide could be released from the peroxisomes/glyoxysomes or mitochondria for example during photorespiration or oxidation of excess reducing equivalents that have exited the chloroplast (Smirnoff and Arnaud, 2019). Pilati et al. (2014) also speculate that it may signal a temporary switch to fermentative respiration. In any case it likely relies on the activity of a superoxide dismutase (SOD), of which isoforms are present in almost all organelles (Alscher et al., 2002). Up-regulation of a SOD transcript (VIT_214s0030g00950) has been reported at veraison in berries of both Kyoho and its early ripening mutant Fengzao (Guo et al., 2016). Despite considerable differences in ripening time between the two cultivars, the SOD transcript began to accumulate in skins of both cultivars at the initiation of berry softening and peaked at the initiation of berry coloring. It was unclear what type of SOD isoform this gene encoded, although a follow-up study found transcriptional up-regulation of a putative cytosolic Cu/Zn-SOD (Vitvi14g02607) and a putative mitochondrial MnSOD (Vitvi13g00177) in veraison berries of both cultivars (Guo et al., 2020). This work would benefit from more biological replication and expansion to other cultivars, and it would be of great interest to know which organelle generates the hydrogen peroxide that is involved in regulating berry ripening.

The accumulation of ROS is followed by a surge in the appearance of ROS-detoxification transcripts and their cognate proteins including ascorbate and glutathione peroxidases, ascorbate oxidase, catalase, polyphenol oxidase, peroxiredoxins, thioredoxins, glutaredoxins, glutathione-S-transferases, metallothioneins, tocopherol cyclase and lipoxygenases (Jimenez et al., 2002; Pilati et al., 2007, 2014; Negri et al., 2008; Agudelo-Romero et al., 2013; Rienth et al., 2014). During ripening, increases in total glutathione and total ascorbate levels have also been observed as well as an increase in their reduction state (Melino et al., 2009a; Fortes et al., 2011). There is also an increase in the production of galactolipid peroxidation products, directed by lipoxygenase activity (Pilati et al., 2014). The molecular and biochemical changes that occur with the oxidative burst at veraison are indicative of an oxidative stress or “oxidative signaling” event (Foyer and Noctor, 2005) analogous to that of the climacteric burst of tomato fruit (Jimenez et al., 2002).

A negative regulator of transcription, NOR (non-ripening) was recently discovered in tomato, which prevents the transcription of ripening-related genes. The NOR protein is susceptible to post-translational regulation via methionine sulfoxidation (i.e., oxidative damage), causing a loss of DNA-binding capacity and thus enabling the transcription of ripening-related genes (Jiang et al., 2020). Therefore, oxidative stress signals are involved in the initiation of ripening, and such a transcription factor could be explored in relation to grape berry oxidative stress and ripening. A surge in ROS levels in the chloroplasts was also observed during the chloroplast-to-chromoplast transition in the exocarp. The trigger for the oxidative burst in grape berries is currently unknown but it is tempting to speculate that the developmental degeneration of chloroplasts, or chloroplast-to-chromoplast transition (Jimenez et al., 2002; Decros et al., 2019), and diminishing capacity for photosynthesis (Palliotti and Cartechini, 2001) leads to decreased accumulation of Asc and TA at veraison, thus the ascorbate/glutathione antioxidant system becomes overwhelmed, resulting in excess ROS generated from respiration or from photons that can no longer be captured for photosynthesis. As such, it would be expected that decreased Asc (or TA) levels and increased ROS could advance ripening. However, in Micro-Tom tomato fruit lacking GDP-L-galactose phosphorylase (VTC2), low levels of Asc (30% relative to wild type fruit) attenuated the ripening-related peak in H_2_O_2_ and delayed ripening, which was recoverable with the addition of exogenous Asc but further delayed by H_2_O_2_ (Steelheart et al., 2020). Meanwhile ripening of Kyoho grape berries could be brought forward by spraying with H_2_O_2_ (Guo et al., 2019). This underscores the importance, and complicated interplay of Asc and H_2_O_2_ for fruit ripening. Non-enzymatic catabolism of Asc (via DHA) to either OA and threonate (then potentially on to TA, at least in tomato), or to 2,3-diketogulonic acid (and several downstream catabolites) can generate H_2_O_2_ in the apoplast (Green and Fry, 2005; Kärkönen et al., 2017). Therefore, diversion of Asc to TA via the 4,5 cleavage pathway and L-idonate, as seen in grapes, could avert this pro-oxidant characteristic of Asc. Transgenic lines of tomato overexpressing VTC2 resulted in very large increases in Asc (3 to 6-fold) but impaired fruit growth and seed production (Bulley et al., 2012). Therefore a potentially important role for TA may be prevention of Asc or DHA over-accumulation in the apoplast, thus postponing the initiation of ripening until the seed is ready for dispersal. It should be noted, however, that translational repression of VTC2 via an upstream ORF (uORF) may prevent over-accumulation of Asc via a clever negative feedback loop as seen in Arabidopsis, tomato and lettuce leaves (Laing et al., 2015; Broad et al., 2020). If this mechanism is active in fruit cells, TA is less likely to be involved in the moderation of Asc accumulation except perhaps in circumstances where very rapid Asc accumulation requires an immediate overflow valve. A link between the oxidative burst and the abrupt changes in TA biosynthesis and ascorbate/glutathione redox state is hereby tentatively proposed. A higher level of regulation is also likely to occur via crosstalk between hormones including ethylene and ABA (Chervin et al., 2004; Lijavetzky et al., 2012; Böttcher et al., 2013a; Carvalho et al., 2015; Pilati et al., 2017).

### Stress Tolerance

Several studies (many reviewed by Gill and Tuteja, 2010) have used over-expression of DHAR genes from various sources, to improve tolerance of tobacco, potato, and Arabidopsis plants to stresses such as salinity, drought, cold, ozone, heavy metal, herbicide treatment, or H_2_O_2_ application (Chen et al., 2003; Kwon et al., 2003; Chen and Gallie, 2005, 2008; Eltayeb et al., 2006, 2011; Ushimaru et al., 2006; Yin et al., 2010; Chang et al., 2017). Generally, the transgenic leaves demonstrated increased Asc due to recycling and in some cases increased levels of total ascorbate, leading to lower ROS accumulation and improved growth metrics in response to stressors. These studies demonstrate the importance of DHA recycling to Asc for tolerating oxidative stress. A study with tomato found that overexpression of DHAR increased total ascorbate levels in fruit but not leaves (Haroldsen et al., 2011). Redirection of DHA to TA biosynthesis removes the potential for Asc recycling and necessitates continuous biogenesis of Asc in pre-veraison berries. It is unclear whether this would have an impact on the antioxidant capacity of the Asc pool and stress tolerance. Studies of transgenic grapevines or natural mutants with impaired or increased TA accumulation are necessary to determine whether the promotion of TA biosynthesis could increase or decrease stress tolerance of grapevines. An exploration of publicly available datasets (below, Table 1) showed that TA biosynthesis genes are largely unaffected by environmental conditions.

**TABLE 1:** Summary of transcriptomic responses of *VvLidh* and *Vv2kgr* to environmental cues.

Treatment/Condition	Log2 FC		Source
	*VvLidh*	*Vv2kgr*	
Water deficit	−	−	Berdeja et al., 2015
Water deficit	−	−	Catacchio et al., 2019
Cold night temperature	−	−	Sawicki et al., 2019
Elevated light	−	−	du Plessis et al., 2017
Salt (leaves)	−	−	Upadhyay et al., 2018
Salt (leaves)	−	−	Das and Majumder, 2019
Increased source:sink	−	−	Pastore et al., 2011
Copper stress	−	−	Leng et al., 2015
Terroir	−	−	Cramer et al., 2020
Abscisic acid	−	−	Pilati et al., 2017
Abscisic acid	0.08	−0.001	Rattanakon et al., 2016
Water deficit	−0.01 to 0	−0.02 to 0.02	Dal Santo et al., 2016
Water deficit	−0.2 to 0.9	−0.4 to 0.3	Ghan et al., 2015
Water deficit	**0.4 to 1.4 (veraison or ripening berries)**	−	Savoi et al., 2017
Extended drought	**0.6 (veraison berries)**	**0.4 (veraison berries)**	Savoi et al., 2016
Elevated temperature	−0.5 to 1.0 (green or veraison berries)	−	Lecourieux et al., 2017
Elevated temperature	**1.3 (ripening berries)**	−	Lecourieux et al., 2017
Elevated temperature	**−1.4 to −2.1 (green or veraison berries)**	−	Rienth et al., 2016
Elevated temperature	**1.1 (ripening berries)**	−	Rienth et al., 2016
Day (c.f. Night)	**−1.2 (late ripening)**	−	Rienth et al., 2014
Cold, anaerobic storage	**2.8**	−	Maoz et al., 2019

*Dashes indicate datasets where log2-fold data could not be retrieved, as only DEGs were presented. In these cases it is suspected, but not confirmed, that the transcript was unchanged between treatment groups, however it could also reflect limits of detection or data lost during filtering steps. Bold indicates statistically significant differences.*

Transcriptional profiling of all three *VvLidh* genes and the *Vv2kgr* gene with publicly available datasets was difficult due to a lack of microarray probesets and genome accessions for *VvLidh2*, and due to the similarity between *VvLidh1* and *VvLidh3* isoforms such that they may have been indistinguishable in RNAseq experiments. For the purpose of this analysis we assumed that *VvLidh* (VIT_16s0100g00290) represents transcripts of both *VvLidh1* and *VvLidh3*. Most datasets (Table 1) exhibited no changes in expression of *VvLidh* nor *Vv2kgr* (VIT_09s0002g04300), including experiments featuring water limitation (Berdeja et al., 2015; Catacchio et al., 2019), cold night temperature (Sawicki et al., 2019), elevated light (du Plessis et al., 2017), diurnal regulation (Rienth et al., 2014), salt stress in leaves (Upadhyay et al., 2018; Das and Majumder, 2019), increased source-sink ratio via cluster thinning (Pastore et al., 2011), copper stress (Leng et al., 2015) and abscisic acid application (Rattanakon et al., 2016; Pilati et al., 2017). In an experiment reporting the differential terroir effect on Cabernet Sauvignon berries in Bordeaux and Reno, there was also no change, but these experimental samples were skins collected during late ripening so expression of *VvLidh* was likely low anyway (Cramer et al., 2020). In response to elevated temperature exposure there was a small down-regulation of *VvLidh* at green and veraison berry stages (Rienth et al., 2016) and a small up-regulation at ripening stages (Rienth et al., 2016; Lecourieux et al., 2017). There was also a slight up-regulation of both *VvLidh* and *Vv2kgr* transcripts in response to extended drought stress at veraison and during ripening (Savoi et al., 2016), while another water deficit experiment showed up-regulation of *VvLidh* only but was accompanied by a slight increase in TA levels (Savoi et al., 2017). Cold storage of table grapes post-harvest led to an increase in *VvLidh3*, if stored under anaerobic conditions (Maoz et al., 2019). Overall the *VvLidh3* and *Vv2kgr* transcripts are largely unaffected by environmental conditions.

### Flowering Time and Senescence

As reviewed by Foyer et al. (2020), Asc can regulate flowering time via the NO-mediated flowering repression pathway (Kumar et al., 2016), such that application of Asc or L-galactonolactone delays flowering, while knockout mutants of various *Vtc* genes in *A. thaliana* (i.e., less Asc) flower earlier (Kotchoni et al., 2009). Genetic modification of AO led to altered expression and diurnal regulation of catalase genes and photorespiration-related genes in tobacco, as well as altered DHAR and APX activities, MAPK activity and growth response to auxins (Pignocchi et al., 2006). A cytosolic APX knockout in Arabidopsis increased H_2_O_2_ content, delayed flowering, decreased photosynthesis rates, prevented stomatal closure in the dark, and decreased expression of some photorespiration-related genes (Pnueli et al., 2003). Overall, Asc metabolism can regulate many developmental switches including flowering and senescence via altered ROS (or reactive nitrogen species (Podgórska et al., 2017; Smirnoff, 2018; Foyer et al., 2020), while TA biosynthesis could indirectly regulate these by catabolizing Asc in the grape.

## Other Potential Roles of TA Biosynthesis

The high levels of TA in Vitaceae may be considered an evolutionary consequence to aid in the dispersion of the mature seed of the grape berry by birds or animals, ensuring the berry is unpalatable until the seed is mature (Hardie and Obrien, 1988; Hardie, 2000; Brummell et al., 2016). Asc has also long been linked to plant defense against biotic and abiotic stressors (Smirnoff, 2000; Zechmann, 2011). Therefore, the possibility of TA being synthesized and stored in the cell as an alternative defensive mechanism, should also be considered at the whole organism level. The potential role in defense against herbivores was suggested by Böll et al. (2005), who found evidence of calcium tartrate crystals in the midgut of the phloem-sucking grape leafhoppers (*Empoasca vitis*) after feeding on grapevine leaves, with levels increasing during ripening of berries of the vine. Although TA was ineffective at preventing leafhoppers from eating the grapevine leaves, calcium tartrate crystals may have a more toxic effect on other insects that feed on grapevine. Both TA and OA have been identified in the hairs of stinging nettle (*Urtica dioica*) as eliciting a pain response in rats (Fu et al., 2006). The human body, along with rabbits, dogs and rats cannot process TA, which instead must be expelled, or destroyed by microorganisms in the intestinal tract (Underhill et al., 1931; Finkle, 1933; Lord et al., 2005). Nevertheless, TA has been deemed non-toxic and appropriate for use as a food additive (EFSA Panel on Food Additives and Flavourings et al., 2020) although the nutritional benefits have not been fully investigated (Spiller et al., 2003).

TA may be used as a carbon source by some microorganisms. Grape berries infected with *Botrytis cinerea* contained less TA and more sorbitol than uninfected berries (Ribereau-Gayon et al., 2006; Blanco-Ulate et al., 2015), hinting at a negative relationship between these two osmolytes that warrants further investigation considering the *VvLIDH3* is a Class II SDH. Based on transcriptomic data (Blanco-Ulate et al., 2015), infection of Semillon berries with *B. cinerea* led to consistent and significant up-regulation of *VvLidh3* expression in harvest-ripe berries (r = 0.78 between transcript level and measures of rot). However at harvest-ripeness, net TA biosynthesis is expected to be low or negligible. Transcript levels of a Botrytis-encoded tartrate dehydrogenase were also enhanced (Blanco-Ulate et al., 2015), therefore catabolism of TA by the fungus could explain the loss of TA. There were also some changes in Asc biosynthesis genes of the grape (Blanco-Ulate et al., 2015), suggesting that this whole metabolic pathway may be reprogrammed, possibly to elicit an antioxidant response. Meanwhile, transcript levels of *Vv2kgr* were largely unaffected but there were negative correlations (r = −0.77 and −0.79) between measures of rot and the transcript levels of two genes putatively associated with later steps in the TA synthesis pathway, a transketolase and a succinic semialdehyde dehydrogenase. This could offer another explanation for lower TA levels in infected fruit, however these genes are yet to be confirmed as components of the TA biosynthesis pathway. Some yeast strains can also utilize L(+)-tartaric acid as a sole carbon source, including a significant number of Basidiomycetous species (Fonseca, 1992). Basidiomycetes, responsible for white rot (Fischer and García, 2015) have been shown to preferentially degrade the L-isomer over the other isomers (Fonseca, 1992). *Pseudomonas syringae* pv. *Syringae*, responsible for bacterial inflorescence rot (Whitelaw-Weckert et al., 2011), has also been shown to utilize TA (Hall, 2015).

## Identifying New Candidates for the Remaining Steps of TA Biosynthesis

Currently, candidate genes in TA biosynthesis have been mainly identified through a biochemistry-guided approach, i.e., based on their annotated enzymatic functions in catalyzing one of the proposed biochemical reactions in the TA biosynthetic pathway. Other than *Vv2kgr* and *VvLidh3*, no candidates have been identified for the remaining steps of TA biosynthesis. These include the first dedicated step, i.e., the conversion of either Asc or DHA to 2-keto-L-gulonate (which may occur in multiple steps), and the final two steps, i.e., 5-keto-D-gluconate to tartaric acid semialdehyde, and subsequently to TA (Figure 1). It was earlier proposed that a transketolase could cleave 5-keto-D-gluconate into 4C and 2C fragments; its normal role would likely be in the Calvin Cycle or the pentose phosphate pathway but perhaps under certain conditions 5-keto D-gluconate could be a substrate (Salusjärvi et al., 2004). For the final step, we suspect a “tartaric acid semialdehyde dehydrogenase”, which could be a promiscuous succinic acid semialdehyde dehydrogenase or a divergent isoform with tartaric acid semialdehyde as a new preferred substrate. It may also be possible that some of these reactions occur non-enzymatically, or that one enzyme may be responsible for multiple steps, e.g., the *Vv2KGR* could conceivably convert Asc directly to L-idonate, as some activity was observed when the recombinant protein was provided Asc as a substrate (Burbidge, 2011).

To overcome the limitations faced by the biochemistry approach, alternative solutions such as QTL analyses based on bi-parental genetic mapping and GWAS have also been used to identify genetic loci linked to TA production. However, due to the complexity of TA biosynthesis and also the challenges in accurately measuring TA content, many genetic mapping studies using bi-parental crossing populations have failed to identify significant QTLs associated with TA production (Liang et al., 2011; Viana et al., 2013; Chen et al., 2015; Bayo-Canha et al., 2019). Houel et al. (2015), however reported consistent QTLs for TA on linkage groups LG4 and LG7 under multiple environmental conditions. The successful identification of genetic loci in this study may be attributed to the specific parent varieties used, likely to be a critical consideration in designing future QTL studies. In addition to bi-parental mapping studies, GWAS analyses using large germplasm collections have emerged as a promising approach in the search for candidate genes for TA biosynthesis. For example, a recent whole-genome resequencing of 472 *Vitis* accessions allowed comprehensive GWAS analyses on many grapevine traits including TA accumulation (Liang et al., 2019), which identified a putative deacetoxyvindoline 4-hydroxylase (VIT_05s0049g00420) and a cinnamoyl-CoA reductase 1-like protein (VIT_13s0064g00270) as potential candidates related to TA biosynthesis. Noteworthy, deacetoxyvindoline 4-hydroxylase belongs to the oxidoreductase superfamily and catalyzes the oxidation of 2-oxoglutarate (5-carbon) to succinate (4-carbon), thereby potentiating this enzyme in the last two steps of TA biosynthetic pathway, i.e., the conversion of 5-keto gluconate to TA. Experimental evidence is required before we can further speculate on their potential functions in TA biosynthesis. In addition, another comprehensive GWAS analysis of 279 *V. vinifera* L. cultivars also identified loci associated with TA accumulation (Flutre et al., 2020), although at this stage there is insufficient publicly available data to link this to a specific gene or set of genes.

**FIGURE 3:**
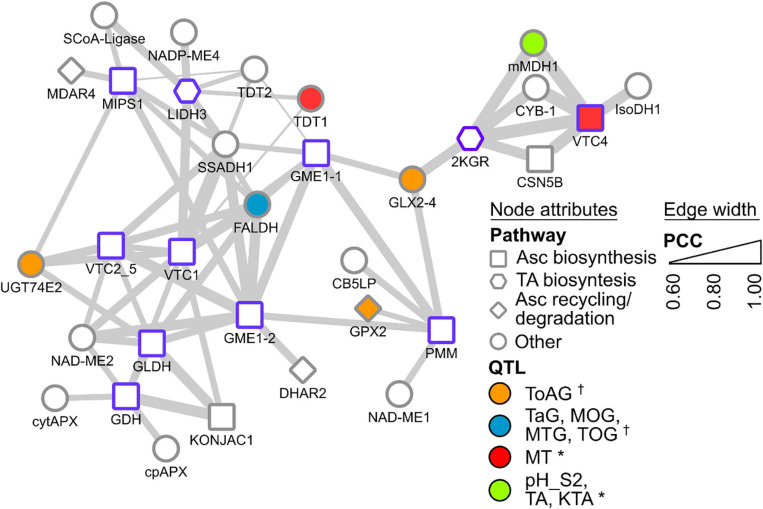
Gene co-expression subnetwork of selected Asc and TA biosynthesis genes. Genes are depicted as nodes and edges depict Pearson correlation coefficients (PCCs) between nodes. Node borders in purple indicate query gene. Node shapes indicate their annotated functional categories. Node color indicate association with known berry acidity QTLs [e.g., †, Houel et al. (2015); *, Duchêne et al. (2020)]. Edge width indicates increasing PCC. Gene abbreviations are SCoA-Ligase succinyl CoA ligase, NADP-ME NADP-dependent malic enzyme, MDAR monodehydroascorbate reductase, MIPS *myo*-inositol-3-phosphate synthase, L-IDH L-idonate dehydrogenase, SSADH succinate semialdehyde dehydrogenase, TDT tonoplast dicarboxylate transporter, GME GDP-D-mannose epimerase, FALDH glutathione-dependent formaldehyde dehydrogenase, VTC1 GDP-D-mannose pyrophosphorylase, VTC2_5 GDP-galactose phosphorylase, NAD-ME NAD-dependent malic enzyme, GLDH L-galactono-1,4-lactone dehydrogenase, GDH L-galactose dehydrogenase, cytAPX cytosolic ascorbate peroxidase, cpAPX chloroplastic ascorbate peroxidase, DHAR dehydroascorbate reductase, UGT74E2 UDP-glucosyltransferase, KONJAC a sugar pyrophosphorylase that can stimulate VTC1 activity, GLX glyoxylase, GPX glutathione peroxidase, CB5LP a cytochrome B5, PMM phosphomannomutase, 2KGR 2-ketogulonic acid reductase, mMDH mitochondrial malate dehydrogenase, CYB Cytochrome B561, CSN5B interacts and modulates activity of VTC1, VTC4 L-galactose-1-phosphate phosphatase, IsoDH isocitrate dehydrogenase. Relevant QTLs from Houel et al. (2015) include: ToAG, total acids at green lag phase; TaG: tartrate at green lag phase; MOG: malate/total acids ratio at green lag phase; MTG, malate/tartrate ratio at green lag phase; and TOG, tartrate/total acids ratio at green lag phase. Relevant QTLs from Duchêne et al. (2020) include: MT, malate/total acids ratio in green berries (Riesling); pH_S2, pH in mid-ripening berries (Gewurztraminer); TA, tartaric acid concentration; KTA, potassium/tartaric acid ratio in berries (Gewurztraminer). For the full lists of co-expressed genes, refer to Wong (2020) supplementary data at https://sites.google.com/view/vtc-agg.

A new approach to identifying candidates for the remaining steps takes advantage of publicly available ‘omics’ data banks and analysis tools. Gene co-expression networks (GCNs) are an emerging resource to study grapevine metabolism (Malacarne et al., 2016; Vannozzi et al., 2018), fruit development/ripening (Massonnet et al., 2017) and stress responses (Savoi et al., 2017; Sun et al., 2018, 2019). Central to GCN analysis is the ‘guilt-by-association’ principle whereby genes that share common functions or related processes are often co-ordinately regulated across a wide range of conditions (e.g., multiple tissues, developmental stages, stress, hormones, etc.). Resources providing customized gene co-expression interrogation are available for grapevine (e.g., Moretto et al., 2016; Wong, 2020). To give an example of how such resources could be used to assist functional gene characterization within grape biosynthetic pathways, we used VTC-Agg (https://sites.google.com/view/vtc-agg) to search for genes that are highly co-expressed with *VvLidh3* and *Vv2kgr*. The data from this database consist of 33 separate experiments including over 1300 different samples across a range of tissues and developmental stages (Wong, 2020). This includes the grapevine transcriptome atlas dataset of Fasoli et al. (2012), which surveyed 54 different sample types, representing vegetative and reproductive organs at numerous developmental stages, including postharvest. The GCN data sets could be mined in multiple ways, including scanning for genes encoding specific proteins of interest such as transketolases and succinate semialdehyde dehydrogenases, or exploring the most highly co-expressed genes (i.e., the top 1% of genes) for other pathways that may occur in parallel with TA biosynthesis, or identify transcription factors or other regulators of the pathway. Surprisingly, *VvLidh3* and *Vv2kgr* were not highly co-expressed with one another based on expression patterns in >1,300 samples (Figure 3), despite showing strong developmental similarities (Jia et al., 2019). This suggests that other conditions, e.g., stress or hormones, affect the expression of these two genes differently. Interestingly, and encouragingly, the transcript that was most highly co-expressed with *VvLidh3*, belonged to a putative GDP-D-mannose pyrophosphorylase gene (*VvVtc1*), while the most highly co-expressed transcript with *Vv2kgr*, was a L-galactose-1-phosphate phosphatase gene (*VvVtc4*). Both of these gene products are involved in plant Asc biosynthesis (Figure 1), indicating close regulation of Asc and TA biosynthesis pathways. A succinic acid semialdehyde dehydrogenase was linked to *VvLidh3* and is therefore a good candidate for the final step of the TA biosynthetic pathway. This strategy was also extended by integrating data from QTL studies aimed at berry acidity (e.g., Houel et al., 2015; Duchêne et al., 2020), to assist with candidate prioritization (Figure 3). Some interesting candidates that were both highly co-expressed with *VvLidh3*, and associated with berry acidity QTLs included two putative tonoplast dicarboxylate transporters (VIT_00s2188g00010 and VIT_00s0187g00130), which associated with a QTL for berry MA:TA concentration ([MA]:[TA]) on chromosome 10 (Houel et al., 2015) and two glutathione-dependent formaldehyde dehydrogenases, or GSNORs (VIT_07s0005g04600, VIT_07s0005g04610) associated with a major QTL for titratable acidity on chromosome 4 (Duchêne et al., 2020). For *Vv2kgr*, candidates included a L-galactose-1-phosphate phosphatase gene, *VvVtc4* (VIT_10s0405g00030) and mitochondrial malate dehydrogenase (VIT_10s0003g01000), which have been implicated with QTLs for [MA]:[TA] and combined pH, TA concentration and potassium:TA concentration ([K+]:[TA]), respectively on chromosome 10 (Duchêne et al., 2020) as well as a predicted glyoxalase (VIT_05s0102g01180), which aligns with a QTL for total acids at green lag phase (Houel et al., 2015). Therefore this analysis has identified several promising candidates for the regulation of TA accumulation in grapevine.

Co-expression analyses were also conducted with known Asc pathway genes (from enzymatic steps summarized in Figure 1), such as phosphomannomutase (*VvPmm*, VIT_15s0046g03520), GDP-D-mannose pyrophosphorylase (*VvVtc1*, VIT_13s0019g02330), GDP-D-mannose epimerase 1 and 2 (*VvGme1-1*, VIT_05s0020g04510; *VvGme1-2*, VIT_14s0030g02180), GDP-galactose phosphorylase (*VvVtc2_5*, VIT_19s0090g01000) and L-galactose-1-phosphate phosphatase (*VvVtc4*, VIT_10s0405g00030). L-Galactose dehydrogenase (*VvGdh*, VIT_03s0088g01250), L-galactono-1,4-lactone dehydrogenase (*VvGldh*, VIT_08s0007g05710), and *myo*-inositol-3-phosphate synthase (*VvMips1*, VIT_07s0031g00920) demonstrated strong and extensive co-expression among various Asc biosynthetic pathway genes, as well as some co-expression with Asc degradation and recycling pathways. For example, co-expression of (i) *VvGdh* with chloroplastic and cytosolic APX (*VvCpapx* and *VvCytapx*), (ii) *VvGme1-2* with DHA reductase 2 (*VvDhar2*), and (iii) *Vv2kgr* and *VvVtc4* with Cytochrome B561. The latter is very interesting, as it links a TA biosynthesis candidate with an Asc biosynthesis candidate and an Asc-dependent oxidoreductase of the plasma membrane involved in electron transport, supporting the idea of TA biosynthesis in the apoplast. Together with the coordinated expression of *VvLidh3* with *VvVtc1*, and *Vv2kgr* with *VvVtc4*, as well as similar metabolite accumulation profiles, there is likely to be coordinated regulation of Asc and TA biosynthetic pathways, potentiating an efficient flux to the endpoint metabolite, as seen for other metabolic pathways in plants (Winkel, 2004; Obata, 2019).

The tight co-regulation of Asc and TA metabolism pathways may involve regulatory control by hormones. A sizable proportion (ca. 10%) of co-expressed genes (of both Asc and TA genes) in the berry were differentially expressed in response to auxin (Dal Santo et al., 2020). A smaller proportion (ca. 5%) of co-expressed genes were modulated by abscisic acid (Pilati et al., 2017). Interestingly, *VvLidh3* was also significantly upregulated in berries by naphthalene acetic acid three hours post-treatment compared to the non-treated control (Dal Santo et al., 2020). These findings indicate a key role of auxin in the regulation of TA metabolism and co-expressed pathways both directly or indirectly. However, we cannot discount the involvement of other hormones given the lack of genome-wide transcriptome studies pertaining to hormonal regulation (e.g., ethylene, jasmonic acid, salicylic acid). Together, this demonstrates how Asc pathway genes can be effectively leveraged to identify more TA candidate genes (e.g., biosynthetic, regulatory, transporters) compared to the restricted lists when using just *VvLidh3* and *Vv2kgr* as guides. We predict that Asc pathway co-expressed genes that also associate with TA concentration or other acidity-related QTLs will likely be relevant in this endeavor.

## Future Directions for Tartaric Acid Research

Despite being first proposed over 50 years ago (Saito and Kasai, 1969), our understanding of the biochemical and genetic pathways to TA in grapes remains significantly under-developed. Two enzyme candidates have been characterized but definitive (i.e., genetic) evidence of their roles remains elusive. Thus far there are no known genetic nor biochemical regulators of the pathway. While ‘conventional’ molecular and biochemical approaches may in time yield additional candidates, research on TA biochemistry in grapes should be conducted with a mind to alternative possibilities. For example, some parts of TA biosynthesis may occur without the assistance of an enzyme, or a single enzyme may be responsible for multiple proposed steps. Meanwhile, the search for novel proteins contributing to a specialized pathway of TA biosynthesis could be directed instead to enzymes of common metabolic pathways with potential sub-functions, or “moon-lighting” functions. For example, genes that are transcribed in a pattern that is inconsistent with their sequence-predicted functions and more consistent with the transcriptional patterns of *VvLidh3* and *Vv2kgr*, and the timing and localization of TA biosynthesis. As TA accumulates to large quantities very quickly [up to 30% of assimilated CO_2_ in very young berries (Saito and Kasai, 1968; 1969)], candidate proteins are likely to be highly abundant, especially during early development.

An intriguing aspect of TA is the existence of multiple biosynthetic pathways, which have arisen separately in different organisms, despite the limited distribution of this metabolite in plants. In the leaves of Geraniaceae plants Asc is cleaved between C2 and C3, with the C3-C6 fragment giving rise to L-threonate and subsequently converted to TA, and C1-C2 becoming OA (Williams and Loewus, 1978). In grape berries L-threonate and OA also occur via 2,3 cleavage of Asc. However, TA arises from a separate pathway whereby Asc is first converted to 5-keto-D-gulonate before being cleaved between C4 and C5, with C1-C4 going on to become TA (reviewed by Ford, 2012). It is unknown why grapevines enlist an entirely new set of enzymes when the 2,3-cleavage activity for Asc is already active. Perhaps the enzyme(s) responsible for converting L-threonate to TA in Geraniaceae are absent or inactive in Vitaceae. OA accumulation typically, but not always, follows the same developmental pattern of accumulation as TA (Melino et al., 2009a; Simson and Debolt, 2012) and the two coexist in the same cell types, thus the pathways likely compete for Asc or DHA (DeBolt et al., 2004). The different biochemical routes to TA observed between species may also reflect contrasting roles of the acid. Updated radiolabelling experiments utilizing ^13^C-Asc and tandem mass spectrometry could be used to determine the proportion of TA biosynthesis that occurs via the 2,3 and 4,5 cleavage pathways in grapes. Measurement of TA precursors as part of wider ranging metabolomic studies may also assist in confirming the existence of a particular route(s) of TA biosynthesis without the need for radiolabeled precursors, and could identify environmental conditions that are favorable for the biosynthesis of TA precursors. Comprehensive studies taking into account environmental and vintage effects are also essential to understanding and anticipating future physiological consequences for grapevines (Cholet et al., 2016). Above all, transgenic grapevine studies with altered expression of *Vv2Kgr*, *VvLidh3* and any other candidates will be necessary for confirmation of the TA biosynthetic pathway genes. Further investigation into the potential of DHA as the precursor to TA should also be explored via transgenic work in TA-accumulating species and tissues.

The classification of *VvLIDH1* and *VvLIDH3* as “Class II” plant SDHs and *VvLIDH2* as a “Class I” SDH provides an opportunity to more deeply understand these enzymes in grapevine and in other plants. Class I SDHs should be clearly differentiated from Class II SDHs when analyzing their potential function in TA biosynthesis because the latter may be the only “genuine” L-IDH. Special attention should also be given to the clear transcriptional divergence between Class I and Class II SDHs, which may shed light on their different biological functions. While the transcript profiles of *VvLidh1* and *VvLidh3* strictly match the developmental pattern of TA accumulation in the berries, a characteristic that was key to the original functional characterization of *VvLidh3* (DeBolt et al., 2006), the Class I SDH (*VvLidh2*; VIT_16s0100g00300) transcripts actually increase from veraison (Sweetman et al., 2012; Conde et al., 2015). Therefore, the Class II SDH genes have not only diverged with respect to their substrate preference, but also expression pattern. The regulators of confirmed L-IDHs may activate transcription in response to photosynthetic, redox or ROS signals and should be investigated for such responsive elements.

Insights could also be gained from other species such as rose-scented geranium, where secondary structures have been predicted for Asc and TA biosynthesis enzymes, including substrate binding sites of L-IDH (Narnoliya et al., 2018). Some important residues included Cys47, His72, Glu73, Glu158, consistent with those identified in *VvLIDH3* for substrate binding Cys36, His61, Glu62, Glu147 (Jia et al., 2015). Predictions of isoelectric point and other functional properties were also carried out in geranium (Narnoliya et al., 2018), followed by the identification of non-coding RNAs that may regulate expression of the gene (Narnoliya et al., 2019). Such information could assist functional analysis, mutagenesis and regulation studies of *V. vinifera* L-IDH. In addition to grapevine and geranium, although at a less significant level, TA production has also been reported in potato (Galdón et al., 2010), citrus fruits (Nour et al., 2010), and pear (Hudina and Stampar, 2000; Sha et al., 2011). These three species have been shown to contain a single copy L-IDH or “Class II” SDH (Jia et al., 2015). Of particular interest was potato, which as an annual herb plant may have an advantage over other species to be exploited as a model for the identification of other TA pathway genes. On another interesting note, TA has also been identified as the main acid in avocado (Pedreschi et al., 2019) and tamarind (Van den Bilcke et al., 2014) fruits. In contrast to most fruits including grape, avocado contains very low levels of citric and malic acids (Pedreschi et al., 2019). These species would be useful models to investigate the genetic and metabolic basis of distinct organic acid profiles and to elucidate the metabolic function, if any, of TA in these plants.

The elaborate regulation of Asc metabolism via biosynthesis, recycling and transport pathways, and its fundamental importance to the ascorbate/glutathione antioxidant pathway (Foyer et al., 2020) clashes with the idea that the irreversible catabolism of Asc (or DHA) to TA should occur by chance or as an “incidental” overflow valve. We propose that the catabolism of Asc or DHA to TA is a regulated process. The hypotheses that TA biosynthesis contributes to antioxidant metabolism, ROS avoidance or as a sink for excess ascorbate in pre-veraison berries, and the potential link to oxidative burst at veraison require further investigation. Gene editing experiments with grapevines, or other TA-accumulating models mentioned above, would be highly suited to this purpose, targeting *Vv2kgr* and *VvLidh3* in the first instance but also new candidates that arise from *in silico* and QTL analyses. Measurement of H_2_O_2_ evolution and TA localization in berries of cultivars that harbor different oxygen distribution patterns, such as those reported by Xiao et al. (2018), could be explored, as could the use of plant growth regulators that alter the timing of ripening (Böttcher et al., 2011, 2012, 2013b), or mutants with altered ripening times (Wei et al., 2020), to test the relationship between the oxidative burst and TA/Asc metabolism at veraison. A fleshless grape berry mutant, where TA accumulates to normal levels but the lack of mesophyll results in lower MA content (Fernandez et al., 2006), could also be a useful tool for investigating the dynamics of Asc metabolism and TA biosynthesis in grapes, and to determine whether metabolism in the flesh is required to support the oxidative burst observed in the skins. Eventually, once the entire TA biosynthetic pathway and the genes responsible have been established, integration of these genes into a non-TA-accumulating plant would be a valuable approach to investigate the hypothesized roles of TA in plant metabolism.

As a final thought, the pathway to TA synthesis in grape berries may only persist due to centuries of grapevine cultivation via clonal propagation (therefore a lack of meiotic recombination) and selection for traits beneficial to winemaking processes and wine style rather than those essential for the competitive survival of the species. In this way, human activity has cemented the relevant biochemical activities and molecular regulators into the genetic lineage of *V. vinifera*. It is another example of artificial selection of a trait considered favorable by humans but otherwise biologically useless to the host, such as early flowering time, larger seed size and determinate habit in grain crops (Izawa, 2007; Shomura et al., 2008; Tian et al., 2010), increased milk production in dairy cattle (Flori et al., 2009) or appearance and temperament characteristics in dog breeds (Akey et al., 2010). In these examples, analysis of SNPs between breeds, cultivars, progeny and progenitors has shed light on genetic components of these domesticated traits. Such an approach may also be possible for TA accumulation in grapevines, considering the absence of TA in at least one species (DeBolt et al., 2006). In any case, this unusual metabolic endpoint with its favorable acid property likely influenced the initial adoption of grapes for winemaking, as early as 5000 BC (McGovern et al., 1996) and remains a significant player in grape and wine biochemistry today. Now the technology is available to begin manipulating TA levels in grapes and wine, with an aim to improve the quality of products for both industry effectiveness and consumer preference.

## Author Contributions

CS and CAB formulated the fundamental structure and content of the manuscript. CF, VM, DW, YJ, CJ, and KS contributed significant ideas, text, and corrections. SC, PD, MR, CB, RW, and FF provided further useful discussions, comments, and corrections. All authors contributed to the article and approved the submitted version.

## Conflict of Interest

The authors declare that the research was conducted in the absence of any commercial or financial relationships that could be construed as a potential conflict of interest.
