# Erratum: Biosynthesis and Cellular Functions of Tartaric Acid in Grapevines

**DOI:** 10.3389/fpls.2021.728277

**Published:** 2021-07-09

**Authors:** Frontiers Production Office

**Affiliations:** Frontiers Media SA, Lausanne, Switzerland

**Keywords:** grape, fruit, tartaric acid, metabolism, gene, enzyme, antioxidant

Due to a production error, the incorrect email address was presented for the Corresponding Author Crystal Sweetman in the originally published article. The correct email address is: “crystal.sweetman@flinders.edu.au”.

The original article has been updated.

